# Proactive Handover Decision for UAVs with Deep Reinforcement Learning

**DOI:** 10.3390/s22031200

**Published:** 2022-02-05

**Authors:** Younghoon Jang, Syed M. Raza, Moonseong Kim, Hyunseung Choo

**Affiliations:** 1 Department of Electrical and Computer Engineering, Sungkyunkwan University, Suwon 16419, Korea; jang0h@skku.edu (Y.J.); s.moh.raza@skku.edu (S.M.R.); 2 Department of IT Convergence Software, Seoul Theological University, Bucheon 14754, Korea

**Keywords:** Unmanned Aerial Vehicles (UAV), Deep Reinforcement Learning (DRL), Proximal Policy Optimization (PPO), handover decision, mobility management

## Abstract

The applications of Unmanned Aerial Vehicles (UAVs) are rapidly growing in domains such as surveillance, logistics, and entertainment and require continuous connectivity with cellular networks to ensure their seamless operations. However, handover policies in current cellular networks are primarily designed for ground users, and thus are not appropriate for UAVs due to frequent fluctuations of signal strength in the air. This paper presents a novel handover decision scheme deploying Deep Reinforcement Learning (DRL) to prevent unnecessary handovers while maintaining stable connectivity. The proposed DRL framework takes the UAV state as an input for a proximal policy optimization algorithm and develops a Received Signal Strength Indicator (RSSI) based on a reward function for the online learning of UAV handover decisions. The proposed scheme is evaluated in a 3D-emulated UAV mobility environment where it reduces up to 76 and 73% of unnecessary handovers compared to greedy and Q-learning-based UAV handover decision schemes, respectively. Furthermore, this scheme ensures reliable communication with the UAV by maintaining the RSSI above −75 dBm more than 80% of the time.

## 1. Introduction

Unmanned Aerial Vehicles (UAVs) are now increasingly used in industries such as agriculture, entertainment, logistics, and surveillance due to their high speed, maneuverability, and agility. A consistent and good network connection is necessary for successful and persistent operation in all these application, and it requires an efficient mobility management scheme. However, cellular networks are designed to provide services to ground User Equipment (gUE), not the other way around. In 5G mobile networks, the cells have much smaller footprint which increases the signaling cost due to frequent handovers and makes it difficult to maintain functional connectivity with UAVs. Additionally, 3D movements of UAVs at high speed are different than the movement patterns and speeds of gUEs. These characteristics of cellular networks and UAVs make mobility management a challenging task, and require a handover decision scheme for UAVs that takes these characteristics into account.

The selection of a target Base Station (BS) for gUE in conventional mobility management is based on the RSSI value of the BSs. Once the difference between received RSSI from current and target BSs crosses a defined threshold, where the RSSI of the target BS is higher, the handover to the target BS is performed [1]. This conventional handover mechanism is suitable for gUE because the main beams of the cellular antennas are directed towards the ground, and the transition of signal strength from one cell to another is relatively smooth. In contrast, weak and inconsistent lobs of side beams provide intermittent network coverage to UAVs [2] that causes ping-pong handovers [3] under conventional handover mechanism. To provide continuous stable connectivity under these limited conditions, the handovers in UAVs to the optimal target BS must be executed at the appropriate time.

The above-mentioned challenges of a conventional handover were confirmed by a recent study in which the authors analyzed UAV handovers under various speeds and altitudes [4]. Their results showed that the handover failure rate increased with increases in the speed and altitude of the UAV. Similarly, the handover probability with changing altitude values was measured through probabilistic geometric analysis in [5]. These studies confirm the influence of speed and altitude on UAV mobility management and the importance of UAV mobility models such as Straight Line (SL), Random Walk (RW), and Random Way Point (RWP) for designing UAV mobility [6]. Analysis of UAV mobility with respect to various factors is well covered in recent studies, however, there are only few schemes presented to improve the handover performance in UAVs.

The handover performance was improved in a study that used a model-free Reinforcement Learning (RL) algorithm to dynamically tilt BS beams to improve the signal strength to the UAVs [7]. The SL mobility model used in this study does not capture the real environment in which a high-degree tilt of beams is required for high-altitude UAVs, which may affect the service of terrestrial UEs. Instead of altering the network to improve the handover performance, a Q-learning-based handover decision scheme (QHD) for UAVs was presented in Chen et al. [8] that used RW mobility model with constant UAV speed and altitude. The RL handover decision algorithm aims to reduce the number of handovers and improve signal quality, which are two conflicting objectives, and limits the performance of the algorithm with joint increment in the weight values. Preliminary results in our previous work [9] showed that better performance is achievable with the single objective of handover reduction.

In this paper, we propose a Deep Reinforcement Learning (DRL)-based UAV handover decision (UHD) scheme that overcomes the limitations in the aforementioned studies. A UHD takes the RSSI of the surrounding BSs and the current state of the UAV as inputs for a Proximal Policy Optimization (PPO) algorithm within a DRL framework to determine the optimal target BS and handover time. A 3D emulation environment for UAV mobility with UHD is implemented in Unity framework [10], and the results confirmed that a UHD improves communication due to a higher handover reduction rate in comparison to those of existing schemes. The UHD achieves this handover reduction rate with a slight RSSI drop but still maintains it in a range for stable and good data rates. Additionally, the efficacy of the PPO algorithm and proposed reward function is highlighted by a three-times faster convergence towards optimal results than the QHD scheme. In summary the major contributions of the proposed UHD are as follows:

- Dynamic optimization of the UAV handover decision through the proposed DRL framework with PPO algorithm determined the moment for UAV handover execution and enabled the UAV to maintain stable communication with high data rates.
- Reduction in redundant handovers with the proposed reward function that learns to ignore RSSI fluctuations and maintain connectivity to increase UAV flight time by conserving energy from reduced handover signaling.
- Accelerated convergence to an optimal handover decision enabled through a simplified reward function and variance control in the PPO algorithm that reduced overall handovers and increased UAV life.
- 3D UAV environment implementation with the DRL framework to evaluate the proposed UHD, and the results showed that UHD outperformed the current greedy approach and QHD by a 76 and 73% reduction in handovers, respectively.

The rest of this paper is structured as follows. Section 2 discusses recent UAV and mobility management related studies along with an explanation of DRL. Section 3 presents the proposed UHD scheme architecture and thoroughly describes the designed DRL framework for UHD. The emulation results are detailed in Section 4, and Section 5 concludes the paper.

## 2. Literature Review and Background

Mobility studies for UAVs are mostly done from two perspectives. The first group of studies discuss mobility of UAVs to provide network services to gUEs, and the second group focuses on handover decisions and triggering mechanisms for UAVs during flight. This section first presents a literature review for these two groups of studies and then describes RL and DRL in detail.

### 2.1. Literature Review

The agility of UAVs enables them to be used in variety of ways to supplement cellular networks or provide an altogether independent network to gUEs [11]. In particular, the communication between vehicles and roadside units suffers from broken links and delays, so a UAV-based relay network was proposed in Khabbaz et al. [12] to improve communication. To compensate for UAV mobility patterns and to determine the path availability, delay, and data rate of a UAV relay network, mobility and analytical models were proposed, and their results showed a 30–55% increase in path availability [12]. Similarly, a UAV-based ad hoc flying network was proposed to the facilitate data-centric Internet-of-Things (IoT) applications with a focus on a distributed routing framework for reliable data delivery [13]. QoS parameters such as UAV velocity, link availability, load capacity, and delay were used in a neuro-fuzzy interference system for efficient path selection and with a mobility model for a routing framework.

UAVs can be used to deploy an independent wireless network for gUE in remote locations or in natural disasters. Base stations are mounted on UAVs that fly over a defined area controlled through a mobility control algorithm [14]. The aim of the control algorithm is to reduce the distance between the mounted base stations and gUEs to achieve line of sight for increasing throughput up to 82%. This study was extended by the same authors, who redesigned the UAV mobility algorithm to improve spectral efficiency [15]. The results showed that spectral efficiency of up to 34% was achieved with a consumer drone moving at minimal speed. An important factor for achieving high data rates between UAVs and BSs is the quality of the aerial wireless channel. A channel model based on constructive and destructive interfaces predicts the aerial wireless channel and its SNR to schedule a subset of gUEs that maximize network use [16]. Testbed results showed a 56% increased throughput of 802.11n-based WiFi hotspot with predicted wireless channel gUE scheduling.

The mobility characteristics of UAVs played an important role in all of the aforementioned studies. To conduct a realistic and practically viable UAV study, it is essential to design UAV mobility models that are close to real mobility patterns. A mobility model for a UAV-mounted base station was studied in [17], where the initial position of the base station was modeled as a Poisson point process, and each UAV base station moved in a straight line in a random direction. This model was inspired by UAV studies in the third-generation partnership project (3GPP), which measures time-varying interference field at gUEs through stochastic geometry and uses it to calculate time-varying coverage probability and data rates [17]. A similar study by the same authors calculated the data rates for gUEs using time-varying interference fields when UAVs moved based on a RWP mobility model [18]. A system-level analysis of UAV base stations based mobile network is conducted using different mobility models in a finite 3D space, where constraints such as small-scale fading for line-of-sight and non line-of-sight propagation, and multi-antenna operations are taken into account [19].

Deep learning (DL)/Machine Learning (ML) approaches have recently achieved promising results for mobility management of gUEs [20,21,22]. In UAVs, a policy gradient based DRL utilizes the RSSI from gUEs to address the mobility management of UAV base stations for improved data rates in 3D-space [23]. The proposed DRL agent successfully distinguishs between line-of-sight and non line-of-sight environments and adjusts UAV speed and altitude accordingly to maximize the data rates under given environmental constraints. In case of UAV mounted base stations, the focus is to improve the communication from UAVs to gUEs, however, for simple UAVs it is important to receive strong and reliable signal from cellular BSs which is challenging due to complex air-to-ground path loss model. A study solves this problem by proposing RL based dynamic adjustment of antenna tilt angles at the BSs [7]. The antennas are tilt upwards to improve connectivity of UAVs, and are tilt downwards to provide the mandatory services to gUEs with good throughput. The results confirm that dynamic tilting of the antennas improve the connection at UAVs and reduce the handovers that otherwise occur due to unstable connectivity [7].

The mobility management and communication protocols in current cellular networks are all designed for gUEs and are not appropriate for UAVs. A recent study shows that frequency of handovers in UAVs increase with the higher altitude and suggests the need for new handover decision mechanism for UAVs for better connectivity [24]. To overcome this problem, the authors in [8] propose a RL based handover decision mechanism based on the RSSI of the next BS but at the same time penalize the RL agent for making the handover. By associating separate weights for handovers and the RSSI of next BS, the RL agent actually tries to minimize the handover while maintaining a high RSSI, which are two conflicting objectives. For this to work, one weight value has to decrease by same magnitude by which the other one increases, but this is not enforced by the system and can cause sub-optimal performance when both weight values are either increased or decreased. In this paper we fixed this problem by using a single handover weight value and showed that it provides better results in comparison to Chen et al. [8].

### 2.2. Deep Reinforcement Learning Background

**Figure 1:**
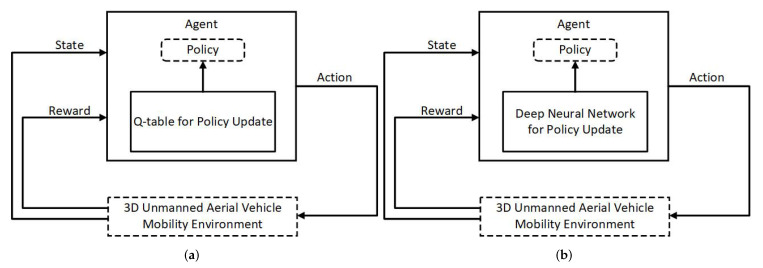
Illustrative comparison between Reinforcement Learning (RL) and Deep Reinforcement Learning (DRL). (**a**) RL with Q-table for policy update. (**b**) DRL with neural network for policy update.

RL is a machine-learning method in which the agent trains by trial and error. A model/algorithm in the agent learns by taking actions on the environment based on its state, as shown in Figure 1. The environment represents the intended system, and the state consists of parameter values that define the environment at the given time. The selection and execution of an action by the agent for a given state of the environment is based on a given policy implemented in the algorithm, and with that result the environment moves to the next state. If the next state moves the environment towards the desired objective, the agent receives a positive reward value calculated through a predefined reward function; otherwise, the agent is penalized with a negative reward value for taking the wrong action. The repetition of this process enables the algorithm to continuously learn to select actions that yield maximum rewards corresponding to the input state. Q-learning [25] is a most widely used RL algorithm that maintains a table of Q-values for all possible actions for corresponding states where Q-values are calculated by using reward values and the Q-function.

The agent in DRL uses a Deep Neural Network (DNN) to optimize its action for a given state of the environment and continuously trains the DNN based on rewards. Multiple DRL algorithms have been proposed over the years, and they can be classified into off-policy and on-policy. Off-policy algorithms are similar to Q-learning and use the epsilon greedy method to explore and update the policy by obtaining a reward value for the action. Deep Q-Network (DQN) [26] and Double Deep Q-Network (DDQN) [27] are the notable examples of off-policy algorithms. DQN uses the Q-network and Q-target DNNs instead of the Q-table to learn from the received rewards to select the action, and these DNNs enable DQN to learn large-scale environments with continuous states. A DDQN resolves the DQN problem of overestimating the Q-value by separately updating the weights of selection (Q-network) and evaluation (Q-target) DNNs.

One limitation of DRL off-policy algorithms is their long convergence time to optimal values, and this is due to the epsilon greedy method and the variance between Q-values. The on-policy algorithms tackle this limitation by using the gradient update method to revise current policy during exploration. Representative examples include the Asynchronous Advantage Actor Critic A3C [28] and Proximal Policy Optimization (PPO) [29] algorithms. The A3C algorithm uses two networks, the advantage value network (critic) and the policy network (actor), to compensate for large variance values. PPO improves upon the fast A3C convergence by proposing a natural policy gradient update by the clip-method, which is computationally complex for real tasks due to second-order optimizations, and the PPO clip method reduces this complexity by computing a natural policy gradient through first-order derivative with soft constraints.

## 3. UAVs Handover Decision with Deep Reinforcement Learning

**Figure 2:**
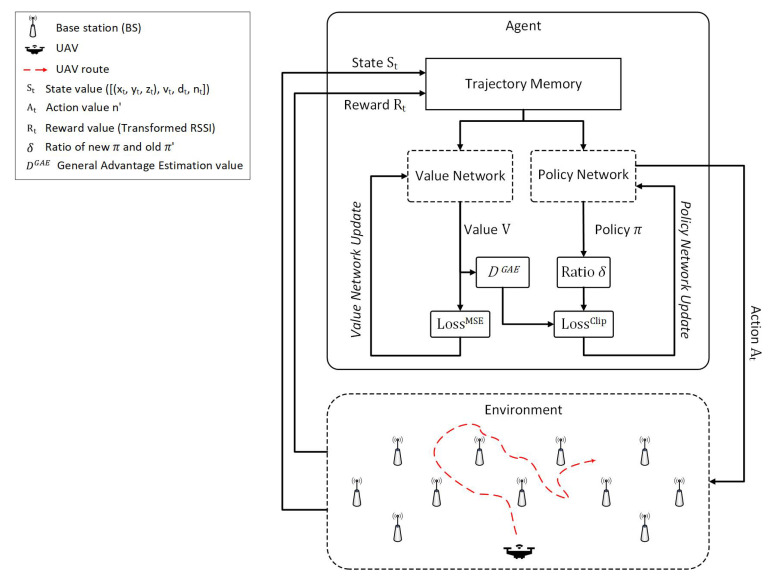
UHD architecture with 3D UAV environment.

The goal of the proposed UHD scheme is to design a handover execution policy that ensures enforcement of necessary handovers at UAVs while maintaining stable connectivity with the ground cellular network. The mobility of UAVs is a three-dimensional (3D) trajectory with variable altitude, and during the flight they perform one or more handovers to maintain communication, as shown in Figure 2. The UHD scheme monitors a received RSSI from BSs at a UAV in a sampling period, and after every period determines whether or not to perform a handover to the target BS with strongest RSSI. In addition to the RSSI, the UHD takes into account several other factors such as location, position, direction, and speed of UAVs while making the handover decisions. In the case of a positive handover decision, the system initiates the control signaling procedure to disassociate the UAV from the current BS and associate it with the target BS. Although the proposed UHD scheme manages handover decision requirements for multiple UAVs, the rest of this paper presents and evaluates a UHD from the perspective of single UAV for clearer understanding and precision.

The conventional RSSI based handover criterion for gUEs works well; however, for a UAV it is notalways suitable to connect to a BS with the strongest RSSI because during the fligh the signal strength of the surrounding BSs abruptly and frequently fluctuates at the UAV [30], resulting in frequent and unnecessary handovers. Moreover, signal strength fluctuations often lead to ping-pong handovers that degrade service quality and cause additional control signaling. One way to solve these issues is to increase the RSSI difference threshold between current and target BSs, but this makes the UAV continue its association with the current BS even after the drop in RSSI value below the feasible communication range. Hence, only threshold adjustment is an inadequate solution, and UAV handover management requires a mechanism that takes into account the variable nature of the network and characteristics of UAV while making handover decisions. The UHD is designed to perform efficient handover decisions that eliminate unnecessary handovers while maintaining the required RSSI for stable communication.

The proposed UHD scheme uses DRL to make optimal handover decisions during a UAV flight to maintain stable and continuous communication while keeping the number of handovers under check. It is worth noting that the signal strength for stable communication is directly proportional to the number of handovers. Therefore, reducing the number of handovers negatively effects the signal strength and requires a delicately designed tradeoff between stable communication and handover frequency. To this end, we designed the objective function of the proposed DRL algorithm so that it flexibly increases or decreases the handover frequency to control the signal quality inversely. This ensures that two conflicting objectives, signal quality and handover frequency, are always inversely related in the objective function and that the system does not simultaneously improve the values of these conflicting objectives. We employed PPO as a DRL algorithm for the learning of the proposed objective function.

PPO is an on-policy DRL algorithm that uses memory to store environment states that are used by value and policy neural networks to train and take an action. In contrast to off-policy algorithms, which use a Q-value to estimate the reward, the value network *V* in PPO accomplishes this reward estimation. The estimated rewards $V(S_{t})$ and $V(S_{t+1})$ from the value network *V* for the input states $S_{t}$ and $S_{t+1}$, respectively, are used along with the reward value $R_{t}$ from the environment to calculate an advantage value (*D*) (5). The value network trains by using the Mean Squared Error (MSE) loss function (6) over *D* which aims to reduce the temporal difference between $V(S_{t})$ and $\gamma V\left(S_{t+1}\right)+R_{t}$, where γ is a discount rate.

(1) $$D_{t}=\gamma V\left(S_{t+1}\right)+R_{t}-V\left(S_{t}\right)$$

As shown in (1), *V* derives $V(S_{t})$ and $V(S_{t+1})$, which are the expected values of the predicted rewards from the input states $S_{t}$ and $\gamma S_{t+1}$, respectively; thus, *V* updates toward minimizing the temporal difference between $V(S_{t})$ and $\gamma V(S_{t+1})$ + $R_{t}$.

(2) $$Loss^{MSE}=\frac{1}{N}\sum_{l=0}^{N-1}D_{t+l}^{2}$$

The policy network π in PPO trains by using the Generalized Advantage Estimator (GAE) [31], a modified advantage-value function ($D^{GAE}$) that reduces the variance of the estimated reward values. In contrast to $D_{t}$, which uses only the discount rate γ, $D^{GAE}$ adds another variable λ to strike a compromise between bias and variance. $D^{GAE}$ is an accumulation of reward values for next *n* states and is calculated by using (3).

(3) $$\begin{array}{cc} D^{GAE}=\sum_{l=0}^{N-1}\left(1-\lambda \right)\lambda^{l}{\hat{D}}_{t+l},\;where & \left({\hat{D}}_{t+N-1}=\sum_{l=0}^{N-1}\gamma^{l}D_{t+l}\right)\\ =\sum_{l=0}^{N-1}{\left(\gamma \lambda \right)}^{l}D_{t+l} \end{array}$$

On-policy based DRL algorithms accumulate rewards at each step and determine the policy that maximizes rewards through a gradient update. However, if the newly discovered reward is an outlier value, the bias is added in the learning network when it is updated to this outlier value. This results in a large error in estimating the general reward, and a reduction in the convergence rate due to the increase in the variance between predicted rewards. To update π and limit the variance of predicted rewards gradually, PPO uses δ (4), which is a probability ratio of each action ($A_{t}$) by stochastic new policy π and stochastic old policy $\pi {}^{\prime }$ in state $S_{t}$.

(4) $$\delta =\frac{\pi (A_{t}|S_{t})}{\pi {}^{\prime }\left(A_{t}|S_{t}\right)}$$

δ and $D^{GAE}$ are used to calculate the loss ($Loss^{clip}$) (5) for the policy network π in PPO. Additionally, δ is used to calculate clip function (6), where hyperparameter ϵ limits the value of δ to either 1−ϵ or 1+ϵ if δ is lower than 1−ϵ or greater than 1+ϵ, respectively. The clip function returns an unchanged value of δ if (1−ϵ)≤δ≤(1+ϵ). This enables the hyperparameter ϵ to set the size of the gradient and facilitate a gradual update of the policy network that results in a faster convergence than from other on-policy algorithms.

(5) $$Loss^{Clip}=E\left[min\left(\delta \times D^{GAE},\;Clip\left(\delta \right)\times D^{GAE}\right)\right]$$

(6) $$Clip=\left\{\begin{array}{cc} \delta \leftarrow (1-\epsilon ) & \mathrm{if}\;\delta <(1-\epsilon )\\ \delta \leftarrow (1+\epsilon ) & \mathrm{if}\;\delta >(1+\epsilon )\\ \delta & \mathrm{otherwise} \end{array}\right.$$

The proposed UHD architecture consists of a PPO agent with trajectory memory and a 3D UAV environment. Since the agent requires environmental UAV data to learn the best handover decision policy, it receives the UAV state and reward from the environment and stores them in the trajectory memory till it is full. The full trajectory memory is transmitted to the policy and value networks as the training data. The value network is trained in the direction of reducing the temporal difference of the expected values from each time. As a result, the value network returns the expected values of the predicted rewards based on the input states, and calculates the advantage values *D* and $D^{GAE}$, whereas the policy network is updated by finding the ratio δ, which is the ratio between the old state-probability policy $\pi^{\prime }$ and the continuously updated stochastic policy π. By this process, the clip method is used for gradual policy network change. Therefore, through policy π, the UHD determines the best target BS for a UAV to execute the handover. The complete training procedure of UHD through the PPO algorithm is described in Algorithm 1.   
**Algorithm 1:** UHD training procedure.
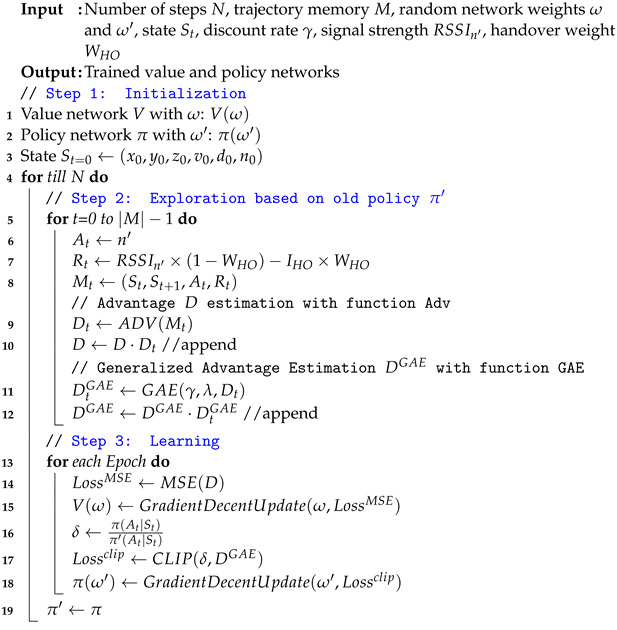


The goal of the PPO algorithm in a UHD is to make handover decisions that reduce the overall number of decisions while maintaining stable communication with the UAV. The value and policy networks in the agent learn based on the state and reward values, which depend upon the actions of the agent. Therefore, we designed state, action, and reward functions in a UHD to encourage a learning framework to execute a handover only when necessary. This not only reduces the overall number of handovers but also curtails the control signaling load on the system. The formal definitions of state, action, and reward in a UHD are as follows.

State: The UAV state at time *t* represents the state space of the environment and consists of 3D positioning $\left(x_{t},y_{t},z_{t}\right)$, velocity $v_{t}$, direction $d_{t}$, and the ID of the currently associated BS $n_{t}$. In addition to RSSI values, state information enables UHD to take probable trajectory and altitude of UAV into account while making handover decision. This helps in decreasing the handovers and mitigates the ping-pong effect. The UAV state is formally defined as

(7) $$S_{t}=\left[\left(x_{t},y_{t},z_{t}\right),v_{t},d_{t},n_{t}\right].$$

Action: The decision to execute the handover defines the action space of a UHD. A positive handover decision implies that the UAV is disassociated from the current BS and has associated with the target BS after completing the standard handover control-signaling procedures in the cellular network. Otherwise, the UAV keeps its association with the current BS. On this account, a positive or negative handover decision can be determined based on the ID of the next BS ($n{}^{\prime }$) from the UHD. In particular, if $n{}^{\prime }$ matches the current BS ID, then it is a negative handover decision; otherwise, it is positive. Accordingly, a UHD action space is defined as

(8) $$A_{t}=n{}^{\prime }.$$

Reward: The UHD learns the desired handover behaviour based on the reward space and takes appropriate action during a UAV flight. The reward space penalizes the UHD agent with a weight $W_{HO}$ for making a positive handover decision ($I_{HO}=1$). At the same time, handovers are necessary for maintaining stable connectivity with the UAV; hence, the UAV agent must get a positive reward for making a necessary handover decision. This requirement is incorporated into the reward space by reducing the weighted signal strength of next BS $\left(RSSI_{n{}^{\prime }}\times \left(1-W_{HO}\right)\right)$ from the initial penalty $I_{HO}\times W_{HO}$. The weighted value $\left(1-W_{HO}\right)$ for $RSSI_{n{}^{\prime }}$ achieves inverse proportionality between the signal strength and handovers and attains a balance between stable UAV connectivity and handover frequency. The reward space can be formally defined as follows:

(9) $$R_{t}=RSSI_{n{}^{\prime }}\times \left(1-W_{HO}\right)-I_{HO}\times W_{HO}\;,$$

where

(10) $$I_{HO}=\left\{\begin{array}{cc} 1 & \mathrm{in}\;\mathrm{case}\;\mathrm{of}\;\mathrm{handover}\\ 0 & \mathrm{otherwise}. \end{array}\right.$$

The UHD reward function uses the weight $W_{HO}$ to reduce dependence on only the RSSI value for a handover decision, so $W_{HO}$ is a penalty for finding a balance between RSSI and handover. For example, a large $W_{HO}$ value in (9) diminishes the influence of the RSSI in a reward calculation, and as a result reduces the number of handovers. Conversely, as the $W_{HO}$ value decreases, the influence of the RSSI increases as a reward, and handovers due to the RSSI increase. This shows that $W_{HO}$ determines the trade-off between stable UAV connectivity and handovers. As a result, the UHD reward function determines the dependence of the RSSI according to the $W_{HO}$ value and learns by receiving a penalty subjected to the presence or absence of a handover, thereby satisfying the balanced handover condition between the two.

**Figure 3:**
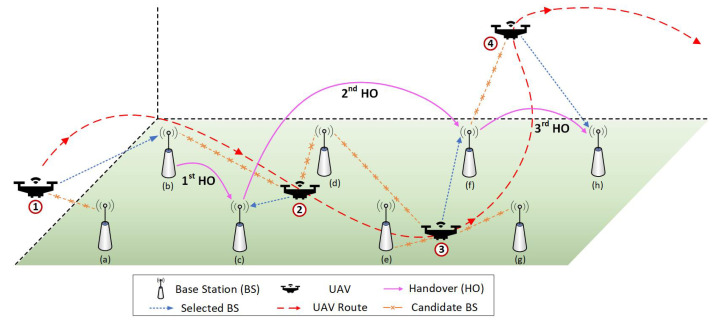
Operational explanation of a UHD with example handovers during a UAV flight.

The overall operation of a UHD for mobility management during a UAV flight is described through an example shown in Figure 3. At position ① in the figure, (a) and (b) are two candidate BSs for initial UAV attachment, and conventional method with greedy algorithm would have selected (a) due to higher RSSI. Whereas, UHD state and reward functions also factor in UAV speed and direction in addition to the RSSI for selecting the target BS, hence, it selects (b) as the target BS. This allows UHD to eliminate an unnecessary handover to (b) later in the case of conventional method. At position ②, the RSSI from BSs (c) and (d) become strong enough to warrant a handover: again, based on UAV position and direction, UHD selects (c) despite the higher RSSI of (d). Similarly, at position ③, the UHD selected (f) as the target BS over BSs (e) and (g) for the same reasons. At the final position ④, UHD uses the RSSI and directional information to understand that that UAV is moving away from BS (f) and executes the handover to BS (h) even though BSs (f) and (g) have similar RSSI values. This demonstrates that the proposed UHD scheme successfully removes unnecessary handovers during a UAV flight and maintains stable connectivity to enable more seamless communication with good data rates. The benefits of UHD include reduced control signaling cost, service disruption, and energy consumption due to the fewer number of handovers, and it is applicable to multiple UAVs in a same network environment.

## 4. Results and Analyses

This section begins with details of the implementation of a 3D UAV mobility environment, and then presents a performance evaluation of a UHD. The handover reduction rate and connection stability are the main criteria for the evaluation against QHD and greedy conventional handover decisions.

### 4.1. Implementation

The evaluation of a proposed UHD scheme requires a 3D UAV environment, and we implemented one using the Unity 3D framework, a C#-based simulator with a game engine that provides 3D- and 2D-development environments. In particular, we developed a hexahedron UHD environment consisting of an area 6×6×0.3 km${}^{3}$ by using the Unity3D 2019.4.13f1 version. Uniformly deployed 45 BSs covered the whole area with no service outages, and the coverage diameter of each BS was defined as 1.5 km per the microcell specification in an urban setting [32]. During the complete flight (trajectory) in the emulated environment, the UAV continuously monitored the RSSIs of the detected BSs and sent a measurement report to the agent to update the handover decision policy.

*Signal strength model*: The UHD environment was developed in a Unity3D framework, which lacks the implementation of network stack and signal propagation models. This meant that the RSSI of BSs at the UAV had to be calculated by implementing a path loss propagation model. In our emulated UHD environment, there are no objects that block UAV line-of-sight communication with the BSs during the complete trajectory. Moreover, the coverage area of BSs overlap with each other in a way that there are no dead zones in UHD environment. This allows us to calculate RSSI by using a simple propagation model and distance (dist) between UAV and BS [33], as defined in (11).

(11) $$RSSI=-10n\log_{10}\left(distance\right)+\alpha ,$$

where *n* is the path loss exponent and its value is set as 2 for a UAV in a UHD environment, and α is the transmitter power with value −10 [33]. During the UAV trajectory, the RSSI was calculated using (11) where the value of dist continuously changed with a change in UAV position (*x*,*y*,*z*) and was measured through: $distance=\sqrt{{\left(x-x_{o}\right)}^{2}+{\left(y-y_{o}\right)}^{2}+z^{2}}$, where $x_{o}$ and $y_{o}$ defined the BS location.

**Figure 4:**
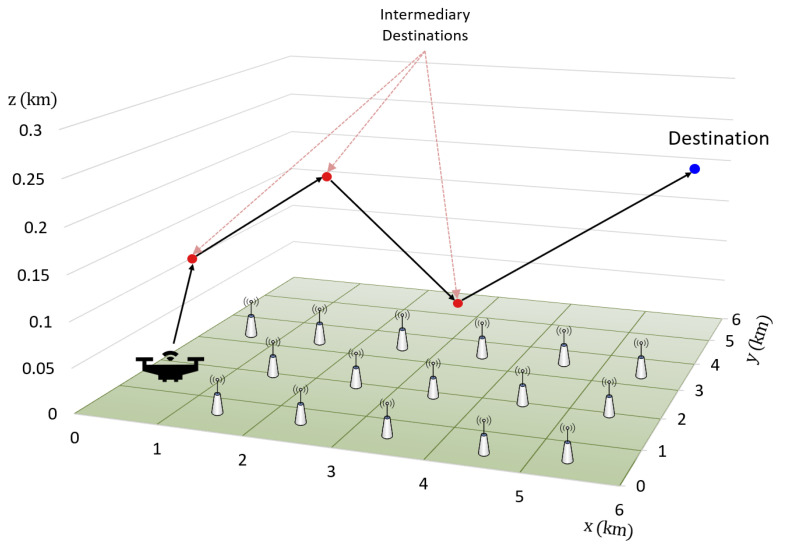
UAV movement in a UHD environment using a Random Way Point (RWP) mobility model.

*Mobility Model*: The proposed UHD scheme enforces policy-based handover decisions on UAVs during flight along the given trajectory. In reality, a UAV trajectory is either predefined or controlled by a remote pilot. However, to implement a trajectory in a UHD environment, the UAV flies from a starting point to a destination using an RWP mobility model [18,34], which requires destination, speed, and direction to be randomly chosen. To implement an RWP, (1) start and destination points of the UAV trajectory are randomly selected in the environment; (2) three intermediate destinations between the starting and destination points are randomly selected, as shown in Figure 4. The UAV flies in a straight line from the starting point to the first intermediate destination (as shown in Figure 4) with a constant velocity in the range of 54–72 km/h based on the average speed of industrial UAVs [35]. Upon reaching the first intermediate destination, the UAV changes direction along the shortest path to second intermediate destination, which also includes altitude variation, and randomly selects the velocity value from the preset range. This process continues till the UAV completes a trajectory by reaching its destination, and then repeats the procedure for different trajectories. It is worth mentioning that the UHD is trajectory agnostic and depends only on the RSSI and current UAV state to make a handover decision; hence, UAV trajectories in a real environment do not affect UHD performance.

The Implementation of signal strength and mobility models enable a UAV to move from initial point to destination and calculate the RSSI during the trajectory. UAV states and rewards based on this mobility and RSSI information are used by a PPO algorithm to learn and update the handover decision policy. The maximum number of UAV states in an experiment are 2,000,000, and the number of states in a trajectory vary depending upon length of the trajectory (i.e., one experiment consisted of many trajectories). The trajectory memory size *M* is defined as 1024, which implies that the value and policy networks are updated once 1024 states are stored in the trajectory memory. The learning rate for gradient update of the networks is 0.0003, and the UHD uses a 0.99 value for both the discount factor γ and variable λ. The hyperparameter ϵ for curtailing the drastic variation in policy generally has a value between 0.1 and 0.3; in our experiment it was set at 0.2. Moreover, Table 1 summarizes the definitions and values of parameters used in the experiments. The results from the UHD scheme were compared with the QHD handover decision policy [8] and the greedy scheme in a conventional handover mechanism.

### 4.2. Results

**Figure 5:**
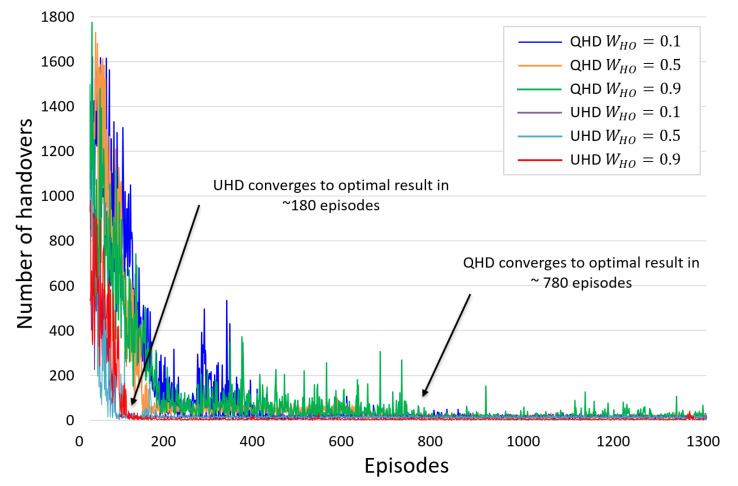
Convergence rate comparison between UHD and QHD methods with handover weight ($W_{HO}$) values 0.1, 0.5, and 0.9.

The networks in a DRL algorithm are generally initialized with random values and require an exploration phase where they learn from the reward values of corresponding actions and converge towards optimal policy. As the exploration happens while the system is online, it is important that this algorithm converge to optimal value as soon as possible. Figure 5 compares the convergence time of a PPO algorithm in a UHD and Q-learning in a QHD scheme [8] for different $W_{HO}$ values in the reward function. The results show that use of single weight value $W_{HO}$ in a UHD avoids the conflicting objectives and enables the PPO algorithm to converge 3.5 times faster than the QHD: the UHD takes about 100 episodes to converge, whereas the QHD takes about 800–1200. Here, each episode represents a single trajectory, and different $W_{HO}$ values show broadly similar convergence rates for both the UHD and QHD. The clipping method in the PPO algorithm controls the wide variations in policy and helps it to converge with a higher rate. Moreover, the convergence and handover decisions of a UHD further improve with multiple UAVs as more data with diverse characteristics is collected in the trajectory memory in a shorter time.

**Figure 6:**
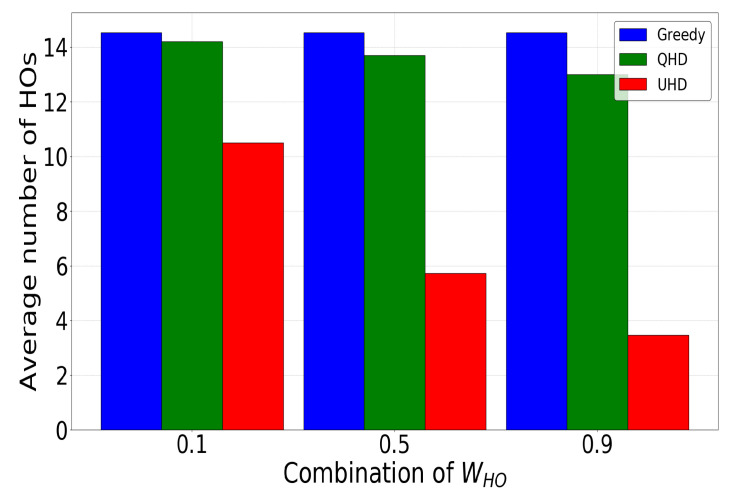
Comparative analysis among UHD, QHD, and greedy methods for the average number of handovers (HOs) in the exploitation phase.

A single UAV handover has an associated handover delay during which communication is stalled due to the exchange of control signaling between the BS and UAV. The occurrence of frequent handovers to maintain high signal quality not only disrupts the communication but also increases the control signaling cost. Hence, reducing the number of handovers to those only necessary results in more seamless UAV communication. To this end, Figure 6 showcases the average number of handovers by a UHD, QHD, and greedy conventional schemes in 30 episodes during the exploitation phase after exploration. The UHD achieved 76% and 73% handover reduction compared to greedy and QHD schemes, respectively, with the highest system penalty for making a handover decision (i.e., $W_{HO}=0.9$). When the handover decision penalty was minimal (i.e., $W_{HO}=0.1$), the UHD performance was much closer to that of the QHD or greedy schemes, and for $W_{HO}=0$ all three schemes had the same performance. The UHD showed a high handover reduction rate with an increase in $W_{HO}$, whereas at the same time the QHD performance stalled due to independent weights for handovers and the RSSI in the reward function. The high RSSI weight value restricted the QHD’s reduction of unnecessary handovers even when the $W_{HO}$ value increased. The results in Figure 6 illustrate this.

**Figure 7:**
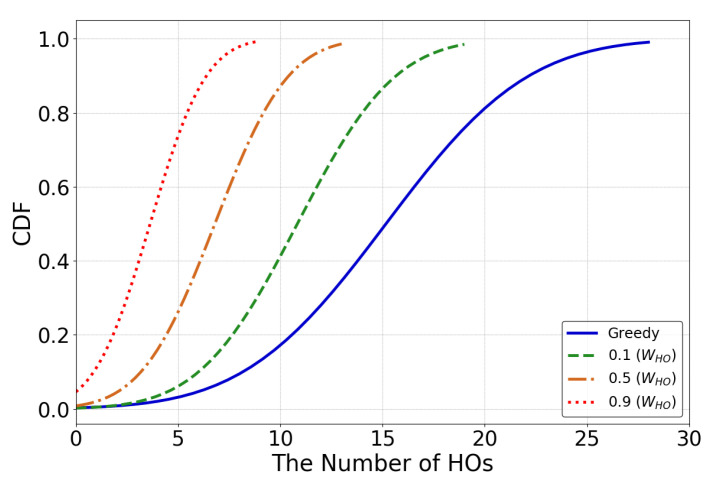
Handovers in a trajectory under greedy and UHD methods with 0.1, 0.5, and 0.9 $W_{HO}$ values.

The average number of handovers showed the overall performance of the UHD, but did not provide detailed insight into UAV handovers in the trajectory. We addressed this by showing a cumulative distribution function of UHD handovers during a trajectory for 0.1, 0.5, and 0.9 $W_{HO}$ values in Figure 7, and compared them with the greedy approach. The results confirmed the findings of Fiigure 6, and showed that the UHD significantly reduced handovers in comparison to the greedy approach when $W_{HO}$ was 0.9. The number of handovers increased and became closer to th egreedy approach when the $W_{HO}$ value decreased to 0.5 and 0.1. These results highlighted the better performance of the UHD reward function, which used a single handover weight value to control the handovers and the RSSI at the UAV. The higher weight value made the UHD maintain the connection with the current BS and compromisef on signal quality, whereas the lower weight value inclined the HD towards attaining a better signal quality by making more frequent handovers. In summary, by eliminating conflicting goals, the UHD enables UAVs to maintain either high signal quality or more seamless communication depending upon the service requirements.

**Figure 8:**
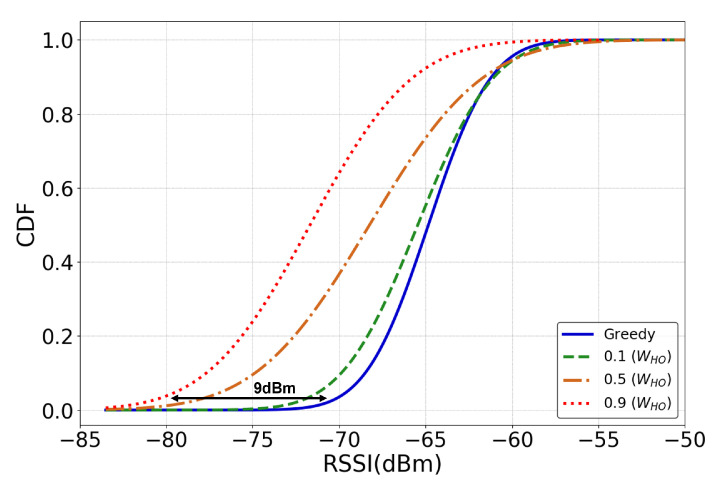
RSSI at the UAV during a trajectory under greedy and UHD methods with 0.1, 0.5, and 0.9 $W_{HO}$ values.

The UHD reduced the handovers in a trajectory by persistently maintaining a connection with the current BS. This could also be perceived as a drawback of the scheme if the connection persists even after the signal quality deteriorated below the stable communication threshold (RSSI < −85 dBm) as shown in Table A1 in Appendix A. To analyze the effects of handover reduction on the RSSI at the UAV, we measured the RSSI of the current BS at the UAV during a trajectory under the greedy scheme and the UHD with $W_{HO}$ values 0.1, 0.5, and 0.9. The results in Figure 8 illustrate that the greedy scheme maintained a good to excellent RSSI (−70 dBm to −60 dBm) at the UAV throughout the trajectory. However, the UHD with a lenient handover curtailing policy ($W_{HO}=0.1$) reduced handovers by 28% while maintaining a good RSSI above −70 dBm for more than 90% of the trajectory. For a more strict handover curtailing policy ($W_{HO}=0.5$), the RSSI at the UAV for most of the trajectory dropped but still remained in the good signal strength range of −75 to −60 dBm for high data rates. The biggest drop in the UAV RSSI comes with the highest reduction in handovers with $W_{HO}=0.9$, but more than 90% of the trajectory RSSI values stayed above the fair signal strength of −80 dBm for reliable communication. From this, it can be concluded that the proposed DRL framework in a UHD successfully achieves the tradeoff between reduced frequency of handovers and signal strength for reliable communication.

**Figure 9:**
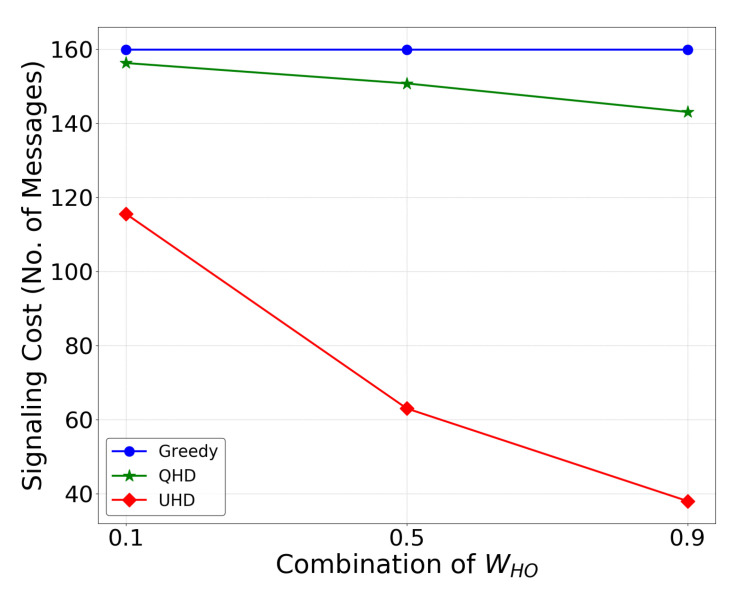
Control signaling cost comparison among UHD, QHD, and greedy methods with 0.1, 0.5, and 0.9 $W_{HO}$ values.

The elimination of unnecessary handovers not only facilitates stable communication but also reduces control signaling costs for the mobile operator. An analytical analysis of handover signaling costs in one trajectory for the UHD, QHD, and greedy conventional method is shown in Figure 9. The cost is calculated by using the number of handovers in a trajectory and the number of signaling messages exchanged between the UAV and BS for a single handover [36]. The results showed that the UHD reduceds the signaling cost for a UAV in a trajectory maximum by 120 and 112% in comparison to the greedy and QHD methods, respectively, with $W_{HO}=0.9$. Even when the handover weight value was low ($W_{HO}=0.1$), the UHD achieveds 32 and 29% lower signaling cost than the greedy and QHD methods, respectively. These results showed that the UHD significantly reduced the network signaling load and reduced energy consumption in UAVs due to fewer transmissions, which increase UAV flight time.

## 5. Conclusions

This paper proposed a DRL-driven UAV handover decision scheme for seamless communication in a 3D environment that reflects the realities of UAVs. In a 3D environment with a RWP movement model based on UAV characteristics, a UHD eliminated unnecessary handovers caused by fluctuating received signal strengths from nearby BSs while maintaining stable communication. In particular, a proposed DRL framework with a PPO algorithm successfully achieved a tradeoff between signal strength and handover frequency. The results showed that use of a single handover weight value enabled the UHD to manage the tradeoff and reduce handovers up to 73% comparing to the QHD method, while maintaining a fair signal strength above −80 dBm. This reduction in handovers resulted in a 29–12% decrease in handover signaling cost comparing to the QHD for $W_{HO}$ values 0.1 and 0.9, respectively. We are currently improving our UAV mobility environment by incorporating more sophisticated wireless transmission models in urban settings and energy consumption models. In the future, this study will be extended to massive UAV fleet scenarios and even to gUE mobility management.

## Figures and Tables

**Table 1:** Parameter definitions and values.

Parameter	Definition	Value
*N*	Total number of UAV states and their corresponding actions during the training	2,000,000
*M*	The trajectory memory size that defines the policy update interval	10,240
η	Learning rate of PPO that determines the gradient step size	0.0003
ϵ	Cut off threshold for difference between old and new PPO policies	0.2
λ	A variable used in the calculation of GAE	0.9
γ	Discount rate for the calculation of GAE	0.9
$I_{HO}$	Handover indicator function	1 or 0
$W_{HO}$	The handover weight value for calculating reward function	0.1, 0.5, 0.9
$R_{t}$	Reward value obtained at step *t*	–
$S_{t}$	UAV state at step *t*	{$\left(x_{t},y_{t},z_{t}\right)$, $d_{t}$, $v_{t}$, $n_{t}$}
$A_{t}$	Action by PPO algorithm at step *t*	Next BS ID ($n{}^{\prime }$)
$(x_{t}$, $y_{t}$, $z_{t})$	Position of UAV at step *t*	(0–6 km, 0–6 km, 0–0.3 km)
$d_{t}$	Direction of UAV movement at step *t*	vector representation
$v_{t}$	UAV speed at step *t*	54–72 km/h

## Data Availability

Not applicable.

## Appendix A

**Table A1:** Relation between RSSI and datarate.

RSSI (dBm)	Signal Strength	Description
−65≤RSSI	Excellent	Strong signal with maximum data speeds
−75≤RSSI<−65	Good	Strong signal with good data speeds
−85≤RSSI<−75	Fair	Fair but useful, fast and reliable data speeds may be attained
−95≤RSSI<−85	Poor	Performance will drop drastically
RSSI<−95	No signal	Disconnection
